# Sensitive and specific detection of mosaic chromosomal abnormalities using the Parent-of-Origin-based Detection (POD) method

**DOI:** 10.1186/1471-2164-14-367

**Published:** 2013-05-31

**Authors:** Joseph D Baugher, Benjamin D Baugher, Matthew D Shirley, Jonathan Pevsner

**Affiliations:** 1 Program in Biochemistry, Cellular and Molecular Biology, Johns Hopkins School of Medicine, Baltimore, MD, 21205, USA; 2 The Johns Hopkins University Applied Physics Laboratory, Laurel, MD, 20723, USA; 3 Program in Human Genetics, Johns Hopkins School of Medicine, Baltimore, MD, 21205, USA; 4 Department of Neurology, Hugo W. Moser Research Institute at Kennedy Krieger Institute, 707N. Broadway, Baltimore, MD, 21205, USA; 5 Department of Psychiatry and Behavioral Sciences, Johns Hopkins School of Medicine, Baltimore, MD, 21205, USA

**Keywords:** Mosaicism, Parent-of-origin, Trio, Parent–child, Software, Microarray, Autism, Cleft, HapMap

## Abstract

**Background:**

Mosaic somatic alterations are present in all multi-cellular organisms, but the physiological effects of low-level mosaicism are largely unknown. Most mosaic alterations remain undetectable with current analytical approaches, although the presence of such alterations is increasingly implicated as causative for disease.

**Results:**

Here, we present the Parent-of-Origin-based Detection (POD) method for chromosomal abnormality detection in trio-based SNP microarray data. Our software implementation, triPOD, was benchmarked using a simulated dataset, outperformed comparable software for sensitivity of abnormality detection, and displayed substantial improvement in the detection of low-level mosaicism while maintaining comparable specificity. Examples of low-level mosaic abnormalities from a large autism dataset demonstrate the benefits of the increased sensitivity provided by triPOD. The triPOD analyses showed robustness across multiple types of Illumina microarray chips. Two large, clinically-relevant datasets were characterized and compared.

**Conclusions:**

Our method and software provide a significant advancement in the ability to detect low-level mosaic abnormalities, thereby opening new avenues for research into the implications of mosaicism in pathogenic and non-pathogenic processes.

## Background

Chromosomal abnormalities, including deletions, amplifications, and uniparental disomy (UPD) events, are a significant cause of Mendelian and complex disorders, as well as a source of benign variation within a population. Technological advancements such as SNP microarrays and next-generation sequencing have dramatically enhanced disease research and diagnosis by improving the ability to detect genomic variation. Along with technological advances, new algorithms for data analysis improve our ability to identify biological aberrations within large datasets. Many algorithms are designed to detect abnormalities that result in copy number variation (CNV), but frequently neglect regions of UPD and somatic mosaicism. Within a population of cells originating from a single zygote, any somatic change results in mosaicism, in which a subset of cells harbors a unique genetic variant. Our understanding of the prevalence and consequences of mosaic abnormalities remains limited, due to the difficulty of detecting alterations in a small subpopulation of cells. Mosaic abnormalities have been implicated in a multitude of disorders, including Alzheimer’s disease, schizophrenia, autism, neurofibromatosis, McCune-Albright syndrome, Duchenne muscular dystrophy, Proteus syndrome, heart, kidney, neuromuscular, and dysmorphic syndromes, as well as cancer [1-5]. Lymphoblastoid cell lines, commonly used for disease research, frequently undergo both the introduction of large mosaic abnormalities and the loss of biological mosaicism due to a tendency toward clonality [6,7].

There are many useful algorithms for abnormality detection in SNP array data. Implementations of segmentation-based approaches applied to B allele frequency (BAF) and log R ratio (LRR) values, including BAFsegmentation [8] and MAD [9], are proficient at detecting abnormalities, including mosaicism, when there are suitably large percentages of abnormal cells. SNPtrio makes use of genotypes from parent–child trios and reports uniparental inheritance, but is generally limited to detection of non-mosaic deletion and UPD events [10]. PennCNV joint is a hidden Markov model (HMM)-based CNV detection tool which is capable of improved detection using parent–child trios, but is not designed to detect UPD or partial copy number states [11]. Other HMM-based approaches, including PSCN, genoCN, MixHMM, and GPHMM, can detect CNVs and UPD events in tumor/normal mixtures and are thus capable of detecting mosaic changes at a certain level of resolution [12-15]. A Bayesian-based algorithm, gBPCR, also reports successful detection of both CNVs and UPD events in mixed tumor/normal samples, but has a very long run time (~2 days per sample) [16]. A highly sensitive method for quantification of the level of mosaicism has been reported, which applies the Distribution Analysis by Fitting Integrated Probabilities method to determine the central tendencies of the BAF band distributions, but there is not currently a feasible implementation for detection of unknown mosaic regions [17]. While many algorithms can detect a subset of mosaic abnormalities, the resolution for low-level mosaicism can be greatly improved.

Here we present an algorithm for highly sensitive and specific detection of mosaic and non-mosaic abnormalities in offspring by employing the Parent of Origin-based Detection (POD) method on SNP array data from a parent–child trio. We also describe an implementation of this algorithm in triPOD (**P**arent-of-**O**rigin-based **D**etection in **tri**os), which includes additional parental contribution-based approaches for abnormality detection. triPOD outperforms current state of the art detection methods, shows greatly improved sensitivity for detection of low-level mosaicism, and uniquely provides the parental origin for each detected abnormality. triPOD software is made available as a command line program and as a web application.

## Results

**Figure 1:**
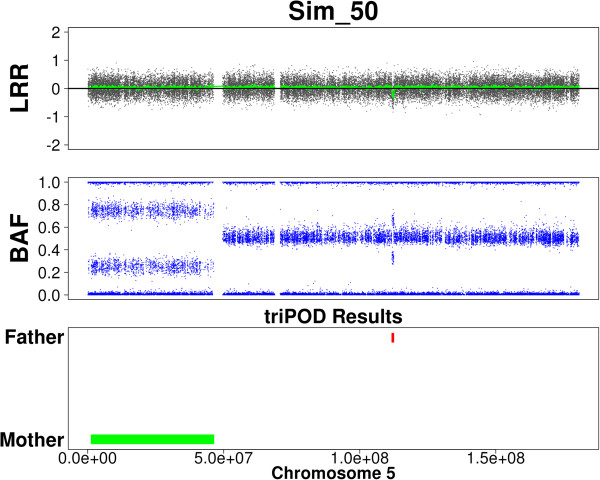
**triPOD graphical output.** triPOD provides graphical output for each chromosome harboring a detected abnormality. The output includes three panels: LRR, BAF, and triPOD Results. The figure illustrates regions of abnormal paternal and maternal contribution: a large maternally contributed mosaic UPD region is plotted in green and a mosaic deletion of paternal origin in red.

SNP array technology provides a convenient source of data for the detection of chromosomal abnormalities. SNP arrays consist of immobilized allele-specific probes for hybridization with fluorescently-labeled target DNA. For the Illumina platform, the normalized intensity ratio at each position is subjected to linear interpolation based on AA, AB, and BB reference genotype cluster positions [18]. The resulting value is an expression of the intensity ratio in terms of the B allele and is referred to as the B allele frequency (BAF). Genotype determination incorporates the proximity of the BAF and LRR values of a sample to those of the reference cluster. When a chromosomal abnormality is present, the BAF values (and often the LRR) deviate from the expected range and possibly affect the genotype call. A SNP with a BAF or LRR value not located within close proximity to an allelic cluster cannot be assigned a genotype and is thus labeled a No Call (NC). A mosaic abnormality can result in a mixture of genotypes (e.g. 80% AB, 20% BB). The resulting diploid genotype as called by the default Illumina algorithm is dependent on the extent of mosaicism and would be either the genotype of the largest subpopulation (e.g. AB) or a NC. Mosaic abnormalities can be visualized as aberrations from the expected genotype bands in a BAF plot (Figure 1, center panel).

### The POD method

The POD method identifies SNPs which are informative for abnormal parental contribution. We define an informative SNP as a polymorphic position where the comparison of parental and progeny genotypes (called or inferred) potentially reveals abnormal parental contribution for the surrounding region (Table 1). Abnormally contributed SNPs are enriched for by analyzing the outliers of the sample-specific normal heterozygous BAF distribution. For example, if at a given SNP position the parental alleles are AA (paternal) and BB (maternal), the genotype of the child is expected to be AB. Thus the BAF value is expected to be a member of the distribution of normal heterozygous BAF values. In this case, if the child’s BAF value is abnormally depressed, it may indicate an underlying biological abnormality resulting in an elevated proportion of paternal A alleles. Conversely, if the child’s BAF value is abnormally elevated, it may indicate an underlying biological abnormality resulting in an elevated proportion of maternal B alleles. The combination of parental genotypes and progeny BAF outliers allows for a highly sensitive inference of mosaic parental contribution regardless of the progeny genotype call. The information content provided by the POD method can be effectively utilized for abnormal region detection by multiple algorithmic and statistical approaches and is the most useful when used concurrently with segmentation or model-based CNV detection. This method can also be used to detect abnormalities in any member of the parent–child trio using inheritance-based expectations of genotype and BAF values (Table 2).

**Table 1:** Informative SNPs for progeny abnormality detection with the POD method

**Father**	**Mother**	**Child BAF**	**Potentially abnormal contribution**
AA, AB, NC	BB	↓	Paternal
AB,BB, NC	AA	↑	Paternal
AA	AB, BB, NC	↑	Maternal
BB	AA, AB, NC	↓	Maternal

Combinations of genotypes and outlier BAF values provide information content useful for the POD method. Given specific parental genotype combinations, when the child’s BAF value is an outlier of the normal BAF distribution, it may be indicative of a departure from expected biallelic inheritance. The ↑ symbol indicates an unexpected elevation of the child’s BAF value (unexpectedly high proportion of B alleles). The ↓ symbol indicates an unexpected depression of the BAF value (unexpectedly low proportion of B alleles).

**Table 2:** Informative SNPs for parental abnormality detection with the POD method

**Child**	**Parent 1**	**Parent 2 BAF**
AB	BB	↑
AB	AA	↓
AA	AA, AB, NC	↑
BB	AB, BB, NC	↓

The POD method can also be employed to detect parental abnormalities. Given specific genotype combinations between a parent and child, when the other parent’s BAF value is an outlier of the normal BAF distribution, it may be indicative of a departure from expected biallelic inheritance. The ↑ symbol indicates an unexpected elevation of the BAF value (unexpectedly high proportion of B alleles). The ↓ symbol indicates an unexpected depression of the BAF value (unexpectedly low proportion of B alleles).

### triPOD description

triPOD is a fast, efficient, multi-threaded software program for chromosomal abnormality detection in offspring using SNP array data from parent–child trios. It is implemented as a Perl script and makes use of the R software environment for graphical output. It can be used for single trios or batches. It is designed to distribute analyses of individual chromosome arms to a user-supplied number of processors and perform parallelized single processor analyses of samples in batch mode. For the current implementation, average run times for analyses of a single trio with ~600,000 SNPs were recorded on a Linux server with 2.67 GHz Xeon x5650 processors and range from ~90 seconds on a single CPU to ~35 seconds employing 10 CPU cores (--cores=10). In batch mode, a number of samples equal to the number of CPU cores can be analyzed in parallel in the same amount of time required for a single sample on a single CPU core. The user can specify the use of the four detection algorithms described below. The output includes detailed annotation of detected abnormal regions in tabular format, optional graphical output of chromosomes harboring abnormalities, a file in BED format for use with genome browsers, an optional file reporting calculated parameters and thresholds, and a log file for error reporting.

### triPOD workflow

triPOD includes an implementation of the POD method, in addition to streak-based approaches to homozygous deletion (HD)(PODhd), single Mendelian error (MI1)-based (PODmi1), and cryptic (low information content) region detection (PODcr). The sliding window approach employed combines high resolution and an adequate sampling of the surrounding region. The following is a summary of the triPOD workflow:

1. Sample-specific parameter specifications and probability estimations are calculated.

2. POD method. SNPs are analyzed for information content based on genotype combinations and progeny BAF values.

3. POD region detection. Informative SNPs are analyzed using overlapping windows. The ratios of parental abnormal SNP contributions are evaluated using a two-tailed binomial test along each chromosome. Overlapping abnormal windows are combined and expanded until evidence of a change in contribution is encountered. Remaining normal segments are evaluated for low-level mosaicism.

4. Streak-based region detection. The PODhd, PODmi1, and PODcr algorithms detect abnormalities by identifying statistically significant streaks of abnormal SNPs.

5. The boundaries of detected abnormal regions are refined using an optimized bidirectional cumulative sums (CUSUM) approach applied to the LRR values or mirrored BAF (mBAF) values (BAF values reflected about the nonhomozygous BAF mean [8]).

6. Steps 2 – 5 are then repeated, following a refinement of the BAF and LRR parameters, which are generally derived from the normal regions of the local chromosome arm.

7. Overlapping detected regions are combined or spliced and annotated for parent-of-origin, type of abnormality, and inheritance pattern.

8. The abnormal regions can be viewed as tabular and/or graphical output (Figure 1).

### Implementation of the POD method

Genetic abnormalities can be detected using a parental contribution model derived from the principles of inheritance. Normal biparental inheritance results in progeny who share approximately 50% of their genome with each parent. Inheritance of a parental chromosome harboring a mutation (a germline mutation) or the acquisition of a somatic mutation alters the expected 1:1 parental contribution ratio for that region. The triPOD implementation of the POD method is based on a statistical model in which the parental contributions revealed by informative SNPs in regions of biparental inheritance can be viewed as a sequence of Bernoulli trials where the outcomes occur with equal probability. So in a set of *n* informative SNPs, the number *k* that indicate paternal contribution can be viewed as the outcome of the binomial random variable *X*_*n*_ ~ *B*(*n*, 0.5). Thus, a two-tailed binomial test can be applied to identify statistically significant deviations from the expected distribution over a region of the genome, indicating the presence of a chromosomal abnormality.

Informative SNPs are detected using a combination of genotypes and progeny BAF values exceeding a threshold. To specify the BAF thresholds, the mean BAF values of heterozygous and homozygous SNPs in normal autosomal chromosomes for each sample are calculated, and thresholds are defined as a specified number of standard deviations (SDs) of the mean. The default number of SDs for heterozygous SNPs is $\sqrt{2}$ based on Chebyshev's inequality, such that for any BAF distribution, a minimum of 50% of SNPs with normal BAFs will be identified and removed from the analysis as uninformative. Chebyshev's inequality sets a distribution-independent minimum threshold for the percentage of values within *k* SDs of the mean, defined as $1-\frac{1}{k^{2}}$ for all k > 1. An initial search for informative SNPs using autosomal BAF thresholds allows for an observation of the distribution of windows containing different amounts of information content (see Methods: Detection of normal chromosomes).

The informative SNPs are first evaluated using overlapping windows (default = 100 SNPs) in single SNP increments along each chromosome arm. For each window the binomial test is applied and the p-value of the observed outcome of the random variable *X*_*n*_ is calculated. The probability mass function for the binomial distribution is defined to be

$$B\left(k;n,p\right)=\left(\begin{array}{c} n\\ k \end{array}\right)p^{k}{\left(1-p\right)}^{n-k},$$

where $\left(\begin{array}{c} n\\ k \end{array}\right)=\left(\frac{n!}{k!\left(n-k\right)!}\right)$, *n* is the number of trials, *k* is the number of successes, and *p* is the probability of success. The formula for calculating the p-value for the two-tailed binomial test is

$$P\left(k;n,p\right)=\left\{\begin{array}{ll} \sum_{i=0}^{k}B\left(i,n,p\right)+\sum_{i=n-k}^{n}B\left(i,n,p\right), & k<n/2\\ 1, & k=n/2\\ \sum_{i=0}^{n-k}B\left(i,n,p\right)+\sum_{i=k}^{n}B\left(i,n,p\right), & k>n/2 \end{array}\right),$$

where, in our case, *p* = 0.5 and *k* is the number of SNP positions with abnormal paternal contribution out of *n* informative SNPs in a window. The null hypothesis, that the window lies in a region of biparental inheritance, is rejected if the p-value falls below the significance threshold, indicating a chromosomal abnormality within or including the current window.

Each overlapping window which contains a new set of informative SNPs and is not inside of an abnormal region constitutes a new binomial test. Due to the unusual dependency structure created by tests applied to overlapping windows, the most appropriate method of controlling the familywise error rate (FWER) is derived from the field of scan statistics, which focuses on the clustering of events observed while scanning time or space. We first compute the expected number of windows *E*(*W*_*k*_) containing *k* informative SNPs given by

$$E\left(W_{k}\right)=\left(N-k+1\right)\sum_{i=k-1}^{N}B\left(i;N,\frac{w}{M}\right),$$

where *M* is the total number of SNPs, *N* is the total number of informative SNPs, and *w* is the window size [19] (adaptation of Equation 17.4). Then at a given threshold *β*, the probability of making a type I error is given by

$$P_{\mathrm{FWER}}\left(\beta \right)=\sum_{k}\sum_{i=1}^{E\left(W_{k}\right)}B\left(i;E\left(W_{k}\right),\gamma_{k}\right),$$

where

$$\gamma_{k}=\max\left(\left\{P\left(j;k,0.5\right)\;|\;P\left(j;k,0.5\right)\leq \beta \;\mathrm{and}\;j\leq k\right\}\right).$$

Thus we can control the FWER by finding a *β* such that *P*_*FWER*_(*β*) is below the α-value. Since our set of possible p-values is discrete and relatively small, this can be done efficiently, and in our implementation we test the values *β*_*k*_ = *P*(*k*; *k*, 0.5) for increasing *k*. The first *β*_*k*_ satisfying *P*_*FWER*_(*β*_*k*_) < *α* becomes our significance threshold for the individual binomial tests.

When an abnormal window is identified, it becomes the seed of an abnormal region, which is expanded until there is evidence of a change-point in the distribution of parental contributions. The boundary is then retracted to the most recent significantly abnormal window and further retracted to the first and last informative SNPs with contribution from the appropriate parent. The region boundaries are then refined using the bidirectional CUSUM approach (see Boundary refinement).

In an abnormal region, all informative SNPs should show abnormal contribution from the same parent unless a genotyping error has occurred. Most detectable errors in an abnormal region will show up as informative for the opposite parent. Therefore we can view each SNP in the window as a Bernoulli trial for which the probability of success (in this case success is defined as the SNP being informative for the opposite parent) is the estimated error rate *e* (see Methods: Data quality adjustments). As before, we can apply a binomial test to assess deviations from the expected distribution which would indicate that the abnormal region has ended and initiate boundary retraction. In this case the applicable test is one-tailed, and the p-value is equal to $\sum_{i=k}^{n}B\left(i,n,e\right).$

Chromosomal regions without detected abnormalities undergo further analysis using a larger overlapping window size (default = small window ×5). This analysis is specifically designed to enhance the detection of large low-level mosaic abnormalities and illustrates the utility of employing multiple window sizes within the POD method. A large window analysis has higher sensitivity and lower specificity than a small window analysis. By implementing an initial analysis with smaller windows, a large majority of abnormalities will be detected with highly specific boundaries before any remaining low-level mosaic abnormalities would be detected during the subsequent large window analysis. This allows for an elevation of overall sensitivity with a minimal reduction in overall specificity.

### Streak detection

When analyzing trios, there are many additional ways to evaluate parental contribution data. To complement the POD approach, triPOD employs a streak-based algorithm for detection of HD- and MI1-based abnormalities, as well as abnormal regions lacking sufficient parental information content. A streak is comprised of adjacent informative SNPs. Informative SNPs are defined differently depending on the algorithm used for streak detection and autosomal rates are calculated (see Methods: Autosomal rate calculations). The minimum number of adjacent informative SNPs which can be considered a statistically significant abnormality is calculated by computing the minimum *m* for which the p-value of the occurrence of *m* adjacent informative SNPs falls below the user defined α-value. A highly accurate approximation of the p-value of a streak of size *m* is given by

$$P_{s}\left(m;n,p\right)=1-Q_{2}{\left(Q_{3}/Q_{2}\right)}^{\left(n/m\right)-2},$$

where

$$Q_{2}=1-p^{m}\left(1+\mathrm{mq}\right),$$

$$Q_{3}=1-p^{m}\left(1+2\mathrm{mq}\right)+0.5p^{2m}\left(2\mathrm{mq}+m\left(m-1\right)q^{2}\right),$$

*n* is the total number of SNPs, and *p* is an autosomal rate of informative SNPs [19] (Equation 4.9).

For HD detection, since the source of parental DNA is considered an appropriate proxy for the individual gametes comprising the zygote, HD regions in a parent indicate a total absence of genetic contribution and are thus detected and reported as abnormalities in the child. In the PODhd algorithm, an HD SNP is defined as having a LRR value sufficiently outlying the distribution ofsingle-copy deletion values centered near −0.5 (default = < −1.5). For each trio member, the minimum size of a significant HD streak is computed as above with *p* defined as the autosomal rate of HD SNPs. All HD streaks larger than the minimum size are detected. The overlapping streaks are combined and expanded using the bidirectional CUSUM approach applied to the LRR values. Non-HD regions in the child which correspond to a parental HD region are evaluated using the CUSUM approach and spliced into adjacent abnormal regions of different types, when necessary.

In the PODmi1 algorithm, informative MI1 SNPs are defined as all genotype combinations from which a single inheritance error can be inferred, including all informative SNP combinations with an abnormal BAF value which exceeds 5SD of the normal heterozygous BAF mean. The minimum PODmi1 region size and region detection is performed as above with *n* defined as the total number of polymorphic SNPs and *p* as the autosomal rate of MI1 SNPs. PODmi1 detection is very useful for identifying small non-mosaic or high-level mosaic abnormalities frequently below the detection limit of the POD method. It is also useful for resolving instances in which the POD method detects a small abnormality but is unable to completely refine the boundaries (see Overlap and annotation).

We define cryptic regions as having a statistically significant presence of outlier BAF values. The autosomal rate of non-homozygous SNPs with BAF values which exceed 2SD of the normal autosomal mean is calculated. The minimum size of a streak is computed as above with *n* defined as the total number of polymorphic SNPs and *p* as the autosomal rate of outlier BAF values. Windows with a significant rate of outlier BAF values may be due to a local asymmetry in BAF [20], and are thus evaluated by the distribution of BAF outliers above and below the distribution center. A two-tailed binomial test is applied, where *n* is the total number of outlier BAF values and *k* is the minimum number of the upper or lower values. Windows for which the null hypothesis is rejected, given the α-value corrected for number of detected cryptic regions, are evaluated and undergo boundary refinement with unknown parental origin. Parent of origin is assigned, if possible, during the annotation stage.

### Boundary refinement

**Figure 2:**
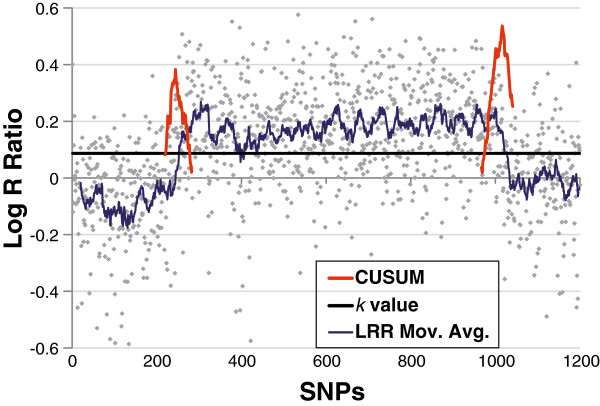
**CUSUM-based boundary refinement.** This figure illustrates the CUSUM-based approach to boundary refinement applied to the LRR values in a large amplification event. The two peaks of the CUSUM plots denote the change-points (boundaries) of the region. The optimized *k* value allows for similar slopes on either side of the detected boundaries (max *S*_*n*_).

The detection of abnormal regions prior to boundary determination requires a unique application of change-point analysis for detection of single change-points bidirectionally from within an abnormality. The cumulative sums (CUSUM) approach is a standard method of change-point analysis developed to detect small changes hidden in a continuous process [21]. A basic one-sided CUSUM equation is

$$S_{n}=\max\left(0,\;S_{n-1}+\left(x_{n}-k\right)\right),$$

in which the change-point is the maximum partial sum *S*_*n*_, *x* is a dataset with *n* members, and *k* is commonly the in-control sample mean or target value. We adapt this equation for boundary detection as follows: *S*_*n*_ is a region boundary defined by a change-point between the normal and abnormal distributions, optimized *k =* (median of the abnormal region – local baseline median)/2, and *x* is a sequence of LRR or non-homozygous mBAF values. Our assignment of *k* maximizes the value of the change-point by creating approximately equal slopes on either side. This estimation of *k* is only optimal when variation between distributions is similar. Since the HD variation is much larger than the other distributions, *k* ≤ 1.5 is considered to be more appropriate. From within an abnormal region the abnormal values will generally be >*k*, creating a positive slope which will peak at the change-point, after which the values in the normal region will generally be <*k*, creating a negative slope (Figure 2).

For an abnormal region to undergo LRR-based CUSUM boundary refinement, the LRR median must meet the LRR threshold requirements (|median| ≥ 0.1 + the local baseline LRR median). If a region does not meet the LRR requirements, it will undergo BAF-based CUSUM boundary refinement if the BAF median meets the BAF threshold requirements (|median| > 0.1 + the local baseline mBAF median). For the application of a one-sided CUSUM approach, the LRR values for regions with a median < 0 are reflected about 0 and a maximum change-point is detected. The members of *x* evaluated by CUSUM are determined as follows:

$$\mathrm{for}\;i=2,\;\left(\begin{array}{l} s_{0}=\mathrm{end},s_{n}\leq \mathrm{start}-10\\ e_{0}=\mathrm{start},e_{n}\geq \mathrm{end}+10\; \end{array}\right\}$$

$$3\leq i\leq 3\min,\;\left(\begin{array}{c} s_{0}=\mathrm{crit}\;|\;\mathrm{cen},s_{n}\leq \mathrm{start}-5i\\ e_{0}=\mathrm{crit}\;|\;\mathrm{cen},e_{n}\geq \mathrm{end}+5i\; \end{array}\right\}$$

$$\;\left(i>3\min,\;\begin{array}{c} s_{0}=\mathrm{start}+\min\;\inf,s_{n}\leq \mathrm{start}-25\\ e_{0}=\mathrm{end}-\min\;\inf,e_{n}\geq \mathrm{end}+25 \end{array}\right\},$$

where *i* = number of SNPs in the abnormal region, *s*_0_ = *x*_0_ and *s*_*n*_ = *x*_*n*_ for the start boundary calculation, *e*_0_ = *x*_0_ and *e*_*n*_ = *x*_*n*_ for the end boundary calculation, *start* = the initial start position of the abnormal region, *end* = the initial end position of the abnormal region, *min* = minimum POD region size, *min inf* = a *min* number of informative SNPs, *crit* = the position of the maximum LRR value in the region which is not equal to *start* or *end*, and *cen* = the position of the center informative SNP for BAF CUSUM. The variables *s*_0_ and *e*_0_ are SNP positions located inside the abnormal region and *s*_*n*_ and *e*_*n*_, respectively, are upstream and downstream baseline SNP positions located outside of the region. The *s*_*n*_ and *e*_*n*_ values are determined by iterative moving median calculations of the data immediately adjacent to the abnormal region boundaries. Median values are calculated for 5*i* ≤ 25 SNPs in overlapping single SNP increments. When the median value is <*k*, it is determined that the window extends into the adjacent baseline data and *s*_*n*_ or *e*_*n*_ = the most distant SNP evaluated. Since a detected region likely includes false positive SNPs in addition to the true abnormality, when 3 ≤ *i* ≤ 3*min*, *s*_0_ = *e*_0_ = the position of the maximum LRR value in the abnormal region to ensure that CUSUM begins within the true abnormality. For BAF-based CUSUM of similar regions, CUSUM begins from the center informative SNP, since extreme values are less informative. In order to reduce the chance that CUSUM finds an incorrect local maximum when evaluating a large region, the analysis begins *min inf* SNPs from *start* and *end* when *i* > 3*min*.

A series of rules are designed to control for unusual situations. If a max peak is not detected, the boundary defaults to the initial value. If the median of the adjacent data is twice as large as or larger than the median of the abnormal region, it is deemed likely that there is an adjacent unique abnormality and the initial boundary remains, unless a CUSUM minimum was detected. Random HD LRR outliers in a hemizygous deletion region are ignored. Random BAF outliers > the region median + 0.1 are ignored.

### Overlap and annotation

For occasions when regions called by different triPOD detection methods overlap, rules have been created to prioritize, combine, and splice such regions. PODhd regions are predominantly small regions and are reported as detected without fragmenting larger overlapping regions. Non-mosaic or high-level mosaic abnormalities can be detected by multiple methods. Since the PODmi1 algorithm is more adept at defining small regions than the POD algorithm, in certain cases we assume that both algorithms are detecting the same small abnormalities and give the PODmi1 region priority. When a POD region overlaps no more than two PODmi1 regions and the information content within non-overlapping POD segments is not larger than the minimum acceptable region size, the POD region is discarded in favor of the PODmi1 region(s). Otherwise, overlapping regions are combined or spliced based upon various factors, including size of region, number of informative SNPs, parental contribution, and type of abnormality.

Detected regions are annotated by parent-of-origin, type of abnormality and inheritance. The type of abnormality is assigned using a threshold-based approach applied to the normalized median LRR value. This value is compared to a threshold normalized to the local baseline LRR median as follows: amplification (AMP) ≥ 0.1, deletion (DEL) > −1.5 and ≤ −0.1, and HD ≤ −1.5. Detected regions with normalized median LRR values > −0.1 and < 0.1 may be any of the following: a UPD region, a region of low-level mosaicism of any type (AMP, DEL, UPD), or a region containing a small abnormality along with many normal SNPs. Within this group, uniparental heterodisomy (UPhD) is assigned to otherwise unannotated regions if the child’s genotypes exactly match the genotypes of the parent of origin, taking into account the estimated error rate. Uniparental isodisomy (UPiD) is assigned to unannotated regions if ≥ 90% of the child’s genotypes are homozygous or if the normalized median mBAF value is > 0.55 and LRR values are > −0.05 and < 0.05. Noisy LRR values may affect proper annotation of small regions. When the inheritance pattern indicates that an abnormality was likely inherited, an annotation of inherited (INH) or inherited with a unique CN state (INH-CN) is provided. The INH state is defined as follows: an AMP is inherited if the contributing parent also has an AMP; a DEL is inherited if the contributing parent also has a DEL; an HD region is inherited if the contributing parent(s) have any heritable combination of HD and DEL regions not indicative of a unique CN state. The INH-CN state is defined as follows: an INH-CN DEL exists when the contributing parent has an HD region and the opposite parent is normal; an INH-CN HD region exists when both parents have a single-copy DEL. We make no judgment as to the inheritance state of any region which does not fall within the above stated parameters.

triPOD provides graphical (Figure 1) and annotated tabular output for detected abnormalities, including parent-of-origin, type of abnormality, inheritance pattern, detection method, region size, number of informative SNPs, and the median mBAF and LRR values for all trio members. The reported regional mBAF and LRR values are normalized as the distance from the median local baseline values. If > 75% of a chromosome arm is abnormal, the medians are normalized to the baseline values of the adjacent chromosome arm. In the case of aneuploidy, the medians are normalized to the autosomal baseline values. Since a parent-of-origin determination is dependent upon the type of abnormality, when the type of abnormality is not called, the detected parental contributor will be designated in the output.

### Benchmarking with simulated data

For benchmarking purposes, we adapted a simulated tumor dilution dataset made available by Staaf *et al*. [8], which has been frequently used for testing the sensitivity and specificity of detection for new algorithms [8,9,12,22] (see Methods: Datasets). This dataset contains 10 simulated abnormalities ranging from 0% to 100% normal cells in intervals of 5%, totaling 21 samples. For use with triPOD, microarray data was obtained for HapMap [23] samples NA06993 and NA06985, the father and mother, respectively, of NA06991, the sample upon which the simulation was constructed. Similar to Staaf *et al*., we assumed that the simulated abnormalities were the only aberrant regions in this dataset.

**Figure 3:**
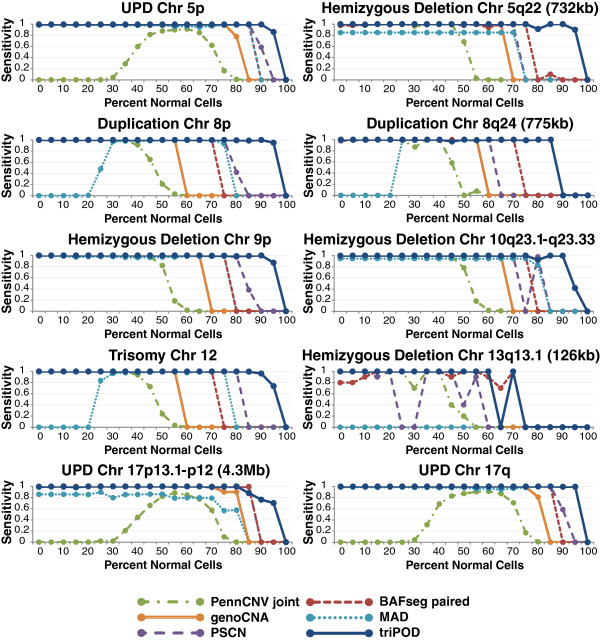
**Sensitivity benchmarking on a simulated mosaicism dataset.** triPOD’s POD implementation was benchmarked against five software programs (paired BAFsegmentation, genoCNA, MAD, PennCNV joint, and PSCN) on a dataset containing 10 simulated abnormalities ranging from 0% to 100% normal cells in intervals of 5%. The sensitivity of detection was calculated for each region. In keeping with previous reports, the mosaic level for each sample was plotted as a percentage of normal cells.

**Figure 4:**
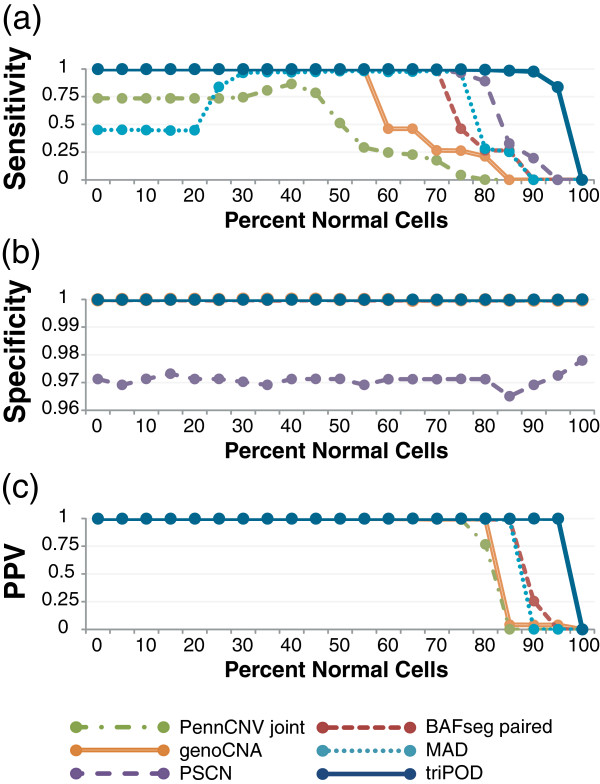
**Mean benchmarking performance statistics.** triPOD’s POD implementation was benchmarked against five software programs (paired BAFsegmentation, genoCNA, MAD, PennCNV joint, and PSCN) on a dataset containing simulated mosaic abnormalities. **(a)** The mean sensitivity, **(b)** specificity, and **(c)** positive predictive value (PPV) are plotted for detection of 10 simulated abnormalities across 21 mosaic states.

The dataset was analyzed using triPOD’s POD method and five leading software programs for chromosomal abnormality detection: paired BAFsegmentation (circular binary segmentation applied to paired tumor/normal samples), genoCNA (a 9 state HMM for CN aberration detection), MAD (Mosaic Alteration Detection, GADA-based segmentation applied to BAF), PennCNV joint (HMM which jointly calls CNVs in trios), and PSCN (a parent-specific copy number segmentation-based HMM algorithm) [8,9,11-13]. Sensitivity and specificity were calculated at each level of mosaicism for each abnormality as in Staff *et al*. [8] (see Methods: Performance calculations). triPOD outperformed all other methods based on sensitivity of detection, displaying a large improvement at low levels of mosaicism (Figure 3). For consistency with previous publications, the level of mosaicism was plotted as a percentage of normal cells, in which case a high percentage of normal cells corresponds to a low-level mosaic abnormality. triPOD’s increase in sensitivity is apparent in all types of simulated abnormalities. The mean sensitivity at each level of mosaicism is presented in Figure 4a. At low levels of mosaicism (85-95% normal cells), triPOD displayed a mean sensitivity of 99%, 98%, and 83%, respectively, compared to the next best performing method (PSCN) with 33%, 20%, and 0%. Mean detection thresholds were calculated as the mean of the percent normal cells (> 50%) at which the sensitivity of detection first drops to zero. triPOD’s mean detection threshold of 96% greatly exceeded all other programs, followed by PSCN (82%) and BAFsegmentation (81%) (Table 3). The high level of consistency displayed by triPOD is evidenced by the fact that 91% of the mosaic abnormalities were detected at a level > = 95% sensitivity, compared to PSCN (79%) and BAFsegmentation (78%) (Table 3). The similarity between a program’s mean detected region size and the mean size of the simulated regions reveals its ability to detect a large abnormality as a single region. Compared to the mean simulated region size of 7355 SNPs, triPOD’s mean region size was 6874 SNPs, BAFsegmentation had 4481, and the others were much smaller (Table 3). The specificity of triPOD, PennCNV, BAFsegmentation, MAD, and genoCNA were comparable and very high (> 0.999), while the specificity for PSCN was much lower (~0.97) (Figure 4b). The reduced specificity of the default PSCN settings allows for an elevation of sensitivity in this comparison. Positive predictive value measurements (Figure 4c) highlight the precision of triPOD’s detection, which is superior to the other programs at low levels of mosaicism, maintaining greater than 0.999 across all levels of mosaicism (excluding 100% normal).

**Table 3:** Sensitivity benchmarking statistics

	**triPOD**	**paired BAFseg**	**PSCN**	**genoCNA**	**MAD**	**PennCNV joint**
Mean detection threshold	**0.96**	0.81	0.82	0.71	0.78	NA
Proportion detected >= 95%	**0.91**	0.78	0.79	0.69	0.37	0.31
Mean Region Size (SNPs)	**6874**	4481	376	579	705	246

Detection statistics are compared between triPOD and other benchmarked programs. The mean detection threshold is the mean of the percent normal cells (> 50%) at which the sensitivity of detection first drops to zero. The proportion detected >= 95% refers to the proportion of the total number of simulated abnormalities detected with >= 95% sensitivity. The mean region size serves as an indication of a program’s ability to call an entire abnormality as a single abnormal region.

### Low-level mosaicism comparisons

**Figure 5:**
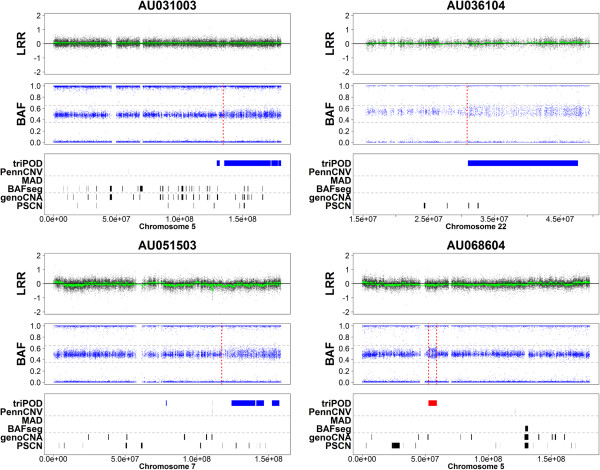
**Detection of low-level mosaic abnormalities in AGRE - Regions 1–4.** triPOD was compared to BAFsegmentation, genoCNA, MAD, PennCNV joint, and PSCN for detection of large low-level mosaic abnormalities in the AGRE autism dataset. Plots of Regions 1–4 are shown for samples AU031003, AU036104, AU051503, and AU068604. For each sample, the top panel is a plot of the LRR values with the moving average (25 SNPs) highlighted in green. The middle panel is a plot of the BAF values with dashed horizontal lines at 0.35 and 0.65 to improve visualization of mosaic splitting of the heterozygous BAF band. The red vertical dashed line indicates the region boundary as detected by our CUSUM-based approach. The lower panel is a graphical representation of the regions detected by each of the six programs. Regions detected by triPOD are colored based on parental contribution: blue = paternal contribution, red = maternal contribution, black = abnormal biparental or undetermined contribution.

**Figure 6:**
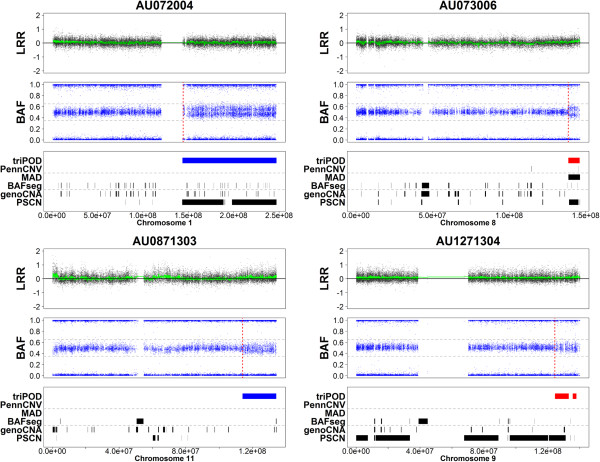
**Detection of low-level mosaic abnormalities in AGRE - Regions 5–8.** triPOD was compared to BAFsegmentation, genoCNA, MAD, PennCNV joint, and PSCN for detection of large low-level mosaic abnormalities in the AGRE autism dataset. Plots of Regions 5–8 are shown for samples AU072004, AU73006, AU0871303, and AU1271304. The plots for each sample are as described in Figure 5.

**Figure 7:**
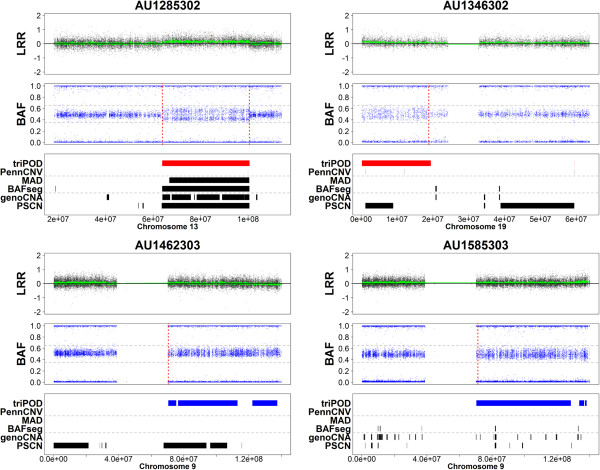
**Detection of low-level mosaic abnormalities in AGRE - Regions 9–12.** triPOD was compared to BAFsegmentation, genoCNA, MAD, PennCNV joint, and PSCN for detection of large low-level mosaic abnormalities in the AGRE autism dataset. Plots of Regions 9–12 are shown for samples AU1285302, AU1346302, AU1462303, and AU1585303. The plots for each sample are as described in Figure 5.

In order to illustrate triPOD’s ability to detect low-level mosaicism in real data, progeny samples harboring large low-level mosaic abnormalities were identified by triPOD in Illumina HumanHap550 trios provided by the Autism Genetic Resource Exchange (AGRE) Consortium [24]. Twelve representative trios were chosen, in which a chromosome in the progeny sample harbored an abnormality which met the following criteria: the change-point is visually identifiable, large (>1000 SNPs), low-level mosaic (estimated < 0.04 deviation of heterozygous BAF values from baseline, which corresponds to < 8.5% mosaicism for UPD events and deletions and < 24% for amplifications), segmental (to aid in graphical visibility), and of reasonable quality (all trio members < 2% NCs). These samples underwent analyses with default parameters using triPOD, BAFsegmentation, genoCNA, MAD, PennCNV joint, and PSCN. For these regions, comparisons between triPOD and the other programs are not strictly benchmarking, since the appropriate regions were first identified using triPOD, by necessity. They serve mainly to illustrate the capabilities of each program for detection of similar regions and to lend real-world support to the results of the simulation analyses. For each region, a CUSUM-based approach applied to a subset of the BAF range (see Methods: AGRE boundary detection) was used to successfully validate the existence of appropriate change-points corresponding to regions wholly or partially detected by triPOD and visually identifiable as a widening or splitting of the heterozygous BAF band. The calculated change-points served as estimates of the region boundaries. A single CUSUM boundary was calculated for terminal abnormalities. Sensitivity was calculated as the ratio of the number of detected abnormal SNPs out of the total abnormal SNPs for each region. A detection threshold was set at a minimum of 10% sensitivity. triPOD dramatically outperformed the other software, based on the number of regions detected and the average sensitivity (91%) (Table 4). PSCN was able to detect 6 of 12 regions, for which the average sensitivity was 69%. MAD was able to detect two of the regions, BAFsegmentation and genoCNA detected a single region, and PennCNV joint was unable to detect any of the low-level mosaic abnormalities. These results are graphically illustrated in Figures 5, 6, 7. Note that several of the programs (BAFsegmentation, genoCNA, and PSCN) called a larger number of abnormal regions in many chromosomes, compared to triPOD, PennCNV joint, and MAD, many of which we assume to be false positives.

**Table 4:** Analysis of large low-level mosaic abnormalities in the AGRE autism dataset

**Region**	**Sample**	**Chr**	**Size(SNPs)**	**% Mosaic**	**triPOD**	**PSCN**	**MAD**	**BAFseg**	**genoCNA**	**PennCNV**
1	AU031003	5	9664	2.1 - 6.4	**0.96**	0.02	0	0.02	0.06	0
2	AU036104	22	4970	4.9 - 12.6	**0.89**	0.02	0	0	0	0
3	AU051503	7	7707	3.7 - 10.7	**0.69**	0.03	0	0	0	0
4	AU068604	5	1499	5.6 - 16.5	**1**	0	0	0	0	0
5	AU072004	1	19981	6.5 - 18.7	**1**	**0.93**	0	0.03	0.05	0
6	AU073006	8	1710	8.4 - 24	**0.97**	**0.87**	**0.99**	0.05	0	0
7	AU0871303	11	5219	2.8 - 8.1	**0.99**	0	0	0.02	0.03	0
8	AU1271304	9	3202	2.5 – 6.8	**0.73**	**0.39**	0	0.01	0.02	0
9	AU1285302	13	7455	7.3 – 21.5	**1**	**1**	**0.93**	**1**	**0.91**	0
10	AU1346302	19	3241	6.7 - 19.2	**1**	**0.45**	0	0	0	0
11	AU1462303	9	15601	2.2 - 6.0	**0.81**	**0.48**	0	0	0	0
12	AU1585303	9	15421	2.8 - 8.1	**0.92**	0.02	0	0.01	0.07	0

The sensitivity of abnormal region detection for 12 samples in the AGRE autism dataset harboring a large low-level mosaic abnormality is presented. The samples were analyzed by triPOD, BAFsegmentation (unpaired), genoCNA, MAD, PennCNV joint, and PSCN. (Sensitivity results < 0.01 are reported as 0). The % Mosaic column is the estimated percent mosaicism calculated, using BAF values >= the baseline median and < 0.7, as the ratio of the distance of the abnormal BAF median from the baseline median and the expected distance of a 100% mosaic abnormality from the baseline median. For low-level mosaicism, the type of abnormality is difficult to distinguish based on LRR values, thus a range is provided to account for regions resulting from a deletion or UPD event (smaller percentage) or an amplification.

### Cross-chip performance: HapMap samples

**Figure 8:**
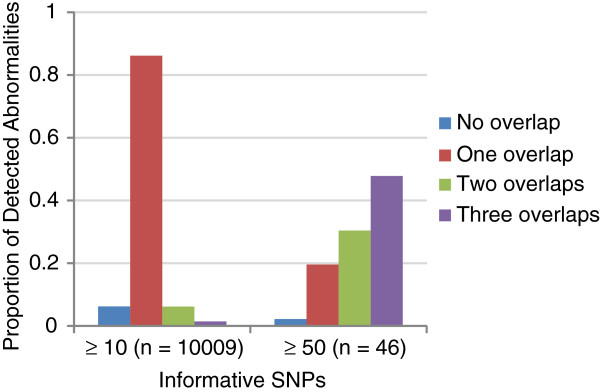
Cross-chip concordance of detected abnormalities.

HapMap CEU and YRI datasets processed on four different Illumina microarray chips and available in the NCBI GEO database [25] were analyzed by triPOD (NC ≤ 5%). Summary results are presented in Tables 5 and 6. Trios which were present in all four datasets and met quality controls were analyzed. The average percentage of NCs ranged from 0.6% to 4.4%. Detected abnormalities were evaluated for overlapping calls (≥ 50% overlap) within the other datasets. The trio including offspring NA10856 was excluded from the overlap analyses due to the emergence of multiple large mosaic abnormalities described below. For abnormal regions with ≥ 10 informative SNPs, 6% of the regions were unique to a single dataset, while 86%, 6%, and 2% overlapped regions called in one, two, or three other datasets. For regions with ≥ 50 informative SNPs, 2% of the regions were unique to a single dataset, while 20%, 30%, and 48% overlapped regions called in one, two, or three other datasets. These results are illustrated in Figure 8. The 660W and Omni1 datasets contain many more detected abnormalities due to the increase in CNV-specific probes (see Table 5). Regions with ≥ 10 informative SNPs in the 660W and Omni1 datasets had 94% concordance. We conclude that, as expected, small regions were much more likely to overlap regions on one similar chip, and that triPOD results for these regions are highly concordant. Also, as expected, large regions were most frequently present in all four datasets and that triPOD showed good concordance given the known fluctuation of mosaic of anomalies in cell lines (highlighted below).

**Table 5:** HapMap CEU datasets

	**1M**	**660W**	**Omni1**	**CytoSNP12**
**Accession**	GSE16894	GSE17208	GSE17197	GSE17123
**# Trios**	27	27	27	27
**Markers**	1128030	634750	1014080	277297
**CNV-specific**	29367	62095	88450	467
**Date Processed**	5/19/2008	2/10/2009	6/29/2009	7/1/2009
**Abnormalities:**				
**AMP**	77	63	84	18
**DEL**	225	248	277	8
**HD**	401	3877	4231	17
**UPhD**	0	0	0	0
**UPiD**	9	13	10	6
**Unk**	159	50	108	20
**Total**	**871**	**4251**	**4710**	**69**

1M: Human1M-Duov3; 660W: Human660W-Quadv1; Omni1: HumanOmni1-Quadv1; CytoSNP12: HumanCytoSNP-12v2-1; Accession: accession identifier in the NCBI GEO database; Markers: approximate number of autosomal markers following conversion; CNV-specific: the number of autosomal CNV-specific probes; Date Processed: the processing date reported in the Illumina final report. *AMP* amplification, *DEL* hemizygous deletion, *HD* homozygous deletion, *UPhD* uniparental heterodisomy, *UPiD* uniparental isodisomy, *Unk* detected abnormalities of undetermined type.

**Table 6:** HapMap YRI datasets

	**1M**	**660W**	**Omni1**	**CytoSNP12**
**Accession**	GSE16896	GSE17210	GSE17203	GSE17126
**# Trios**	29	29	29	29
**Markers**	1128030	634750	1014080	277297
**CNV-specific**	29367	62095	88450	467
**Date Processed**	5/20/2008	2/10/2009	6/30/2009	7/1/2009
**Abnormalities:**				
**AMP**	109	57	98	24
**DEL**	278	231	263	10
**HD**	395	3295	4009	17
**UPhD**	1	0	0	0
**UPiD**	9	4	3	0
**Unk**	113	45	56	15
**Total**	**904**	**3632**	**4429**	**66**

Rows and columns are defined in Table 5.

**Figure 9:**
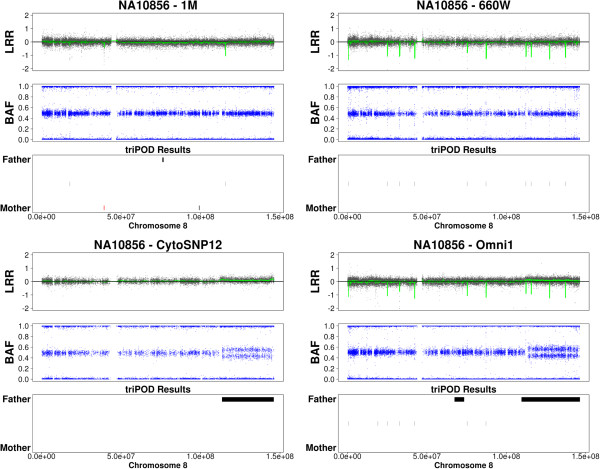
**Emerging mosaicism in HapMap sample NA10856 chromosome 8.** 1M: Human1M-Duov3; 660W: Human660W-Quadv1; Omni1: HumanOmni1-Quadv1; CytoSNP12: HumanCytoSNP-12v2-1.

**Figure 10:**
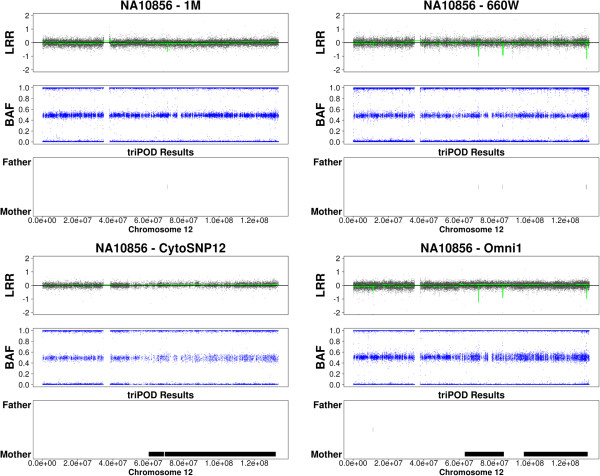
**Emerging mosaicism in HapMap sample NA10856 chromosome 12.** Abbreviations are as defined for Figure 9.

**Figure 11:**
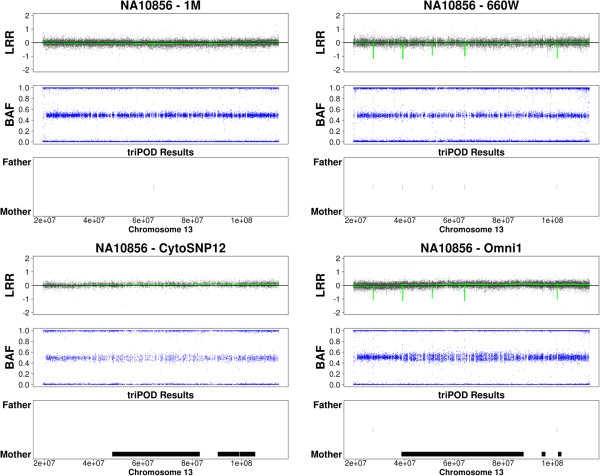
**Emerging mosaicism in HapMap sample NA10856 chromosome 13.** Abbreviations are as defined for Figure 9.

The ability to detect newly emerging mosaic abnormalities is illustrated in Figures 9, 10, 11, in which triPOD results are provided for chromosomes 8, 12, and 13 of sample NA10856. Several large mosaic abnormalities, detected by triPOD and visually identifiable, are present in the Omni1 and CytoSNP12 samples: a large mosaic amplification on chromosome 8qter; a large low-level mosaic abnormality on chromosome 12 qter; a large low-level mosaic abnormality on chromosome 13q. As presented in Table 5, the 1M and 660W samples were processed at earlier dates than the Omni1 and CytoSNP12 samples. We believe that these large abnormalities occurred during passaging and expansion of this cell line and were thus present and detectable only in the more recently processed samples. triPOD also detected a large very low-level abnormality on chromosome 5q of CEU sample NA07029 (not shown), which was possibly not present in the 660W and 1M samples and below the resolution of the CytoSNP12 sample. Newly emerging large mosaic abnormalities were not detected in the YRI trios, although a previously reported [13,26] mosaic amplification of the entire chromosome 9 in NA19208 was detected in all four datasets.

### GENEVA cleft lip/palate and AGRE samples

The cleft lip/palate dataset is a part of the Gene Environment Association Studies initiative (GENEVA) and described in [27]. It consists of parent–child trios run on the Illumina Human610 Quadv1_B microarray platform. Most of the DNA samples were obtained from whole blood, with limited samples from buccal brush/swap, saliva, mouthwash, and dried blood. Of the 2029 trios, 1962 trios passed quality control before analysis with triPOD. The autosomes of 1930 of those were successfully analyzed by triPOD (NC ≤ 3%).

The AGRE trios were previously run on the Illumina HumanHap550 microarray, which does not contain CNV-specific probes. The DNA for these samples was derived from cultured lymphoblastoid cell lines. The autosomal data of 1587 trios was successfully analyzed by triPOD (NC ≤ 3%).

**Figure 12:**
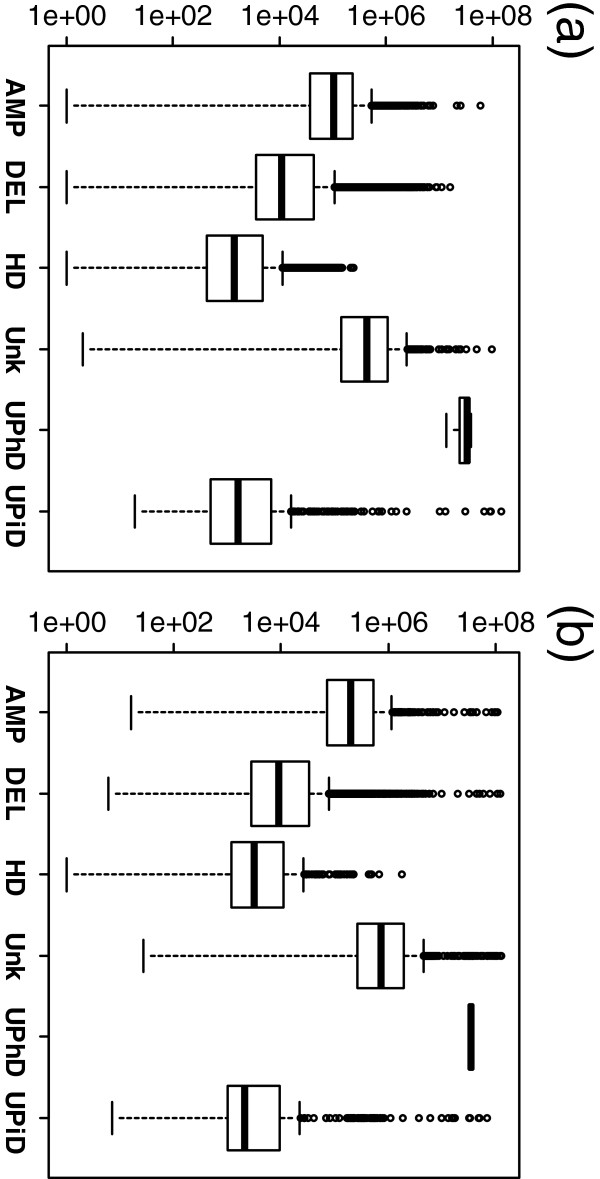
**Size distributions of detected abnormalities.** Boxplots of the sizes (bp) of abnormalities detected in the **(a)** cleft and **(b)** AGRE datasets.

The total numbers and ratios of detected abnormalities by type (AMP, DEL, HD, UPhD, UPiD, Unknown (Unk)) are presented in Table 7. The presence of CNV-specific probes on the Human610 chip used for the cleft data vastly increases the number of detected regions and alters the ratios. The distributions of the sizes for each type of abnormality in the cleft and AGRE datasets are presented in Figure 12. While the medians are similar between datasets, it is evident that the whiskers extend lower for the cleft findings (likely due to small CNV regions) and the outliers tend to be larger and more abundant within the AGRE abnormalities.

**Table 7:** AGRE and cleft datasets

	**AGRE**		**Cleft**	
**# Trios**	1587		1930	
**Markers**	547458		600470	
**CNV-specific**	0		17879	
**Abnormalities:**				
**AMP**	1398	(0.09)	3499	(0.06)
**DEL**	10680	(0.65)	12259	(0.22)
**HD**	2821	(0.17)	37804	(0.66)
**UPhD**	2	(0)	3	(0)
**UPiD**	329	(0.02)	496	(0.01)
**Unk**	1211	(0.07)	3072	(0.05)
**Total**	**16439**		**57130**	

Rows are defined in Table 5. Values in parentheses are proportions of the total abnormalities for each dataset.

For both the cleft and AGRE datasets, the large, highly informative abnormalities were identified. These regions with ≥ 250 informative SNPs were visually investigated and multiple reports for single abnormalities were combined (e.g. each abnormality which encompasses a whole chromosome is expected to be reported as at least two regions since triPOD analysis considers each chromosome arm separately). The resulting regions are presented in Tables 8 and 9. As mentioned above, these large abnormalities occur much more frequently in the AGRE dataset. The proportion of autosomes harboring at least one large informative abnormality in the cleft dataset was 13 of 42460 autosomes (0.0003), while the AGRE dataset contained 49 of 34914 autosomes (0.0014). Thus the AGRE dataset is 4.7 times more likely to harbor large abnormalities than the cleft dataset (p = 6.1e^-8^).

**Table 8:** Large abnormalities in cleft samples

**CIDR_Name**	**Chr**	**Loc**	**Type**	**PO**	**SNPs**	**Inf**	**Det**	**mBAF**	**LRR**	**#Reg**
12254_01	16	pT	UPiD	M	3830	574	MI1	0.51	−0.037	1
12254_01	16	pC,q	UPhD	M	13095	971	POD	0.50	−0.027	2
16194_01	10	qT	DEL	F	2578	431	MI1	0.94	−0.475	1
17008_01	2	W	UPiD	F	49497	6727	MI1	0.87	0.008	2
18113_01	11	qT	DEL	F	1883	337	MI1	0.88	−0.408	1
19004_01	13	qT	AMP	F	13467	1776	POD	0.64	0.240	1
19143_01	18	qT	DEL	F	4150	613	MI1	0.92	−0.521	3
20127_01	11	qT	UPiD	F	16191	2064	POD	0.55	0.004	1
20183_01	13	qT	UPiD	F	21216	2505	POD	0.56	0.022	1
21089_01	20	q	UPiD	M	7420	937	POD	0.58	−0.018	1
21098_01	21	q	UPhD	M	8817	588	POD	0.50	0.003	1
21186_01	17	q	NA	F(C)	10102	422	POD	0.52	0.012	1
21230_01	11	pI	AMP	M	4836	380	POD	0.64	0.227	1
23020_01	18	qT	DEL	F	4358	672	MI1	0.89	−0.614	1

*CIDR_Name* sample identifier from the Center for Inherited Disease Research; *Loc* the location of the abnormality on the chromosome (p=p arm, q=q arm, W=entire chromosome, T=telomeric end, C=centromeric end, I=interstitial); *PO* parental origin (F=paternal origin, M=maternal origin, F(C)=paternal contribution, M(C)=maternal contribution); SNPs=number of SNPs in the detected region; Inf=number of SNPs in the detected region which were informative for the detection algorithm; Det=detection algorithm; #Reg=the number of separate regions reported within a single large abnormality.

**Table 9:** Large abnormalities in AGRE samples

**ID**	**Chr**	**Loc**	**Type**	**PO**	**SNPs**	**Inf**	**Det**	**mBAF**	**LRR**	**#Reg**
AU002503	12	pT	UPiD	F	3882	603	POD	0.73	0.022	1
AU0025312	17	pT	UPiD	F	4221	670	POD	0.61	0.001	1
AU005304	5	W	NA	M(C)	23269	881	POD	0.51	0.017	4
AU016404	6	pT	DEL	M	8574	1365	POD	0.80	−0.385	1
AU016404	15	qT	AMP	F	5964	967	POD	0.62	0.249	1
AU031003	5	qT	NA	F(C)	9497	363	POD	0.51	0.000	4
AU038006	21	W	UPiD	F	8140	722	POD	0.54	0.002	1
AU060704	7	qT	NA	M(C)	14016	508	POD	0.51	−0.095	1
AU070003	11	qT	UPiD	M	14593	2580	MI1	0.82	0.013	3
AU072004	1	q	NA	F(C)	19976	1299	POD	0.53	−0.007	1
AU075208	6	qT	DEL	M	12589	1664	POD	0.67	−0.208	1
AU075208	13	qT	AMP	M	9961	1486	POD	0.59	0.184	1
AU075307	21	W	AMP	M	8250	882	POD	0.65	0.331	1
AU077705	12	qT	NA	M(C)	5016	854	POD	0.65	0.084	1
AU078803	9	W	AMP	M	26873	3833	POD	0.58	0.161	2
AU0871303	5	W	NA	F(C)	33022	1966	POD	0.53	0.067	2
AU0871303	11	qT	NA	F(C)	5178	263	POD	0.52	0.048	1
AU0871303	12	W	AMP	M	26968	1870	POD	0.53	0.150	2
AU0903303	6	W	NA	F(C)	35910	1969	POD	0.52	−0.063	2
AU0924301	12	W	NA	M(C)	27011	2153	POD	0.53	0.073	2
AU0962301	12	W	NA	F(C)	17431	629	POD	0.50	0.000	4
AU0983302	3	pT	UPiD	M	10954	1316	POD	0.58	0.033	1
AU1060301	3	qT	UPiD	M	1890	326	POD	0.66	0.015	1
AU1157303	9	q	UPiD	M	15535	2086	POD	0.57	0.026	1
AU1227303	21	W	AMP	M	8250	553	POD	0.65	0.291	1
AU1227304	12	W	NA	M(C)	26967	2321	POD	0.53	0.057	3
AU1243301	9	qT	AMP	F	5779	419	POD	0.56	0.139	1
AU1277303	12	W	NA	M(C)	16009	649	POD	0.50	−0.036	2
AU1285302	13	qI	NA	M(C)	7510	982	POD	0.54	0.114	1
AU1321301	6	W	NA	F(C)	35956	2604	POD	0.52	−0.069	2
AU1346302	19	pT	NA	M(C)	3301	291	POD	0.53	0.012	1
AU1388302	10	qT	AMP	M	17918	2363	POD	0.59	0.176	1
AU1388302	11	qI	AMP	F	3517	498	POD	0.59	0.093	1
AU1388302	11	qT	DEL	F	10173	1649	POD	0.69	−0.331	1
AU1462301	9	W	NA	F(C)	25921	1688	POD	0.52	0.054	2
AU1462303	6	pT	UPiD	M	9294	1489	POD	0.69	0.014	1
AU1462303	9	q	NA	F(C)	12655	451	POD	0.51	−0.030	3
AU1497301	14	qI	UPiD	M	5337		MI1	1	−0.017	1
AU1497301	14	qI	UPhD	M	9148		MI1	0.52	−0.002	1
AU1497301	14	qT	UPiD	M	3774		MI1	1	0.029	1
AU1529301	19	pT	UPiD	F	2599	374	POD	0.56	−0.005	1
AU1544303	12	W	NA	M(C)	26590	1885	POD	0.52	0.053	2
AU1585303	9	q	NA	F(C)	14175	655	POD	0.51	−0.002	3
AU1590302	6	pT	NA	F(C)	7117	399	POD	0.53	0.010	1
AU1594303	6	W	DEL	M	38044	5388	POD	0.59	−0.132	2
AU1594303	9	W	AMP	M	26276	2372	POD	0.54	0.089	3
AU1601302	12	W	NA	M(C)	26900	1563	POD	0.52	0.056	2
AU1650306	11	qT	NA	F(C)	4955	621	POD	0.55	−0.101	1
AU1650307	9	W	NA	M(C)	26736	2000	POD	0.53	0.070	2
AU1695303	12	W	AMP	F	27318	3128	POD	0.55	0.102	2
AU1791303	9	W	NA	F(C)	26393	1296	POD	0.52	0.056	3
AU1822302	16	p+qC	UPhD	M	14865	1187	POD	0.50	−0.014	2
AU1822302	16	qT	UPiD	M	1866	344	POD	0.91	−0.025	1

Abbreviations as described in Table 8. Note – a large abnormality detected in sample AU1497301 was manually annotated as adjacent regions of UPD.

A large study on the presence of mosaic abnormalities (≥ 50 kb) in GENEVA datasets was recently reported by Laurie *et al*. [28]. Since the cleft dataset consists of parent–child trios, we were able to compare regions detected in the offspring. They reported 10 mosaic abnormalities in 9 offspring samples within the cleft dataset, the identifiers of which were provided upon request by the authors. These findings are presented in Table 10 along with the overlapping triPOD findings. triPOD detected all of the previously reported abnormalities with an average concordance of 99.9%. triPOD also detected a vast number of additional abnormalities, many of which are presumed to be mosaic based on normalized median mBAF values, although triPOD does not attempt to distinguish mosaic from non-mosaic abnormalities.

**Table 10:** **Comparison of offspring abnormalities reported by Laurie *et al*.**[28]

	**Laurie *et al*.**		**triPOD**								
**CIDR_Name**	**ID**	**Type**	**Chr**	**Start**	**Stop**	**Type**	**PO**	**Mb**	**mBAF**	**LRR**	**Concordance**
13069_01	58	loss	17	26056501	27338617	DEL	F	1.28	0.61	−0.166	0.9977
17008_01	330	aupd	2	23012	242692820	UPiD	F	6.08	0.87	0.008	0.9996
19173_01	160	loss	2	165344115	166416787	DEL	F	1.07	0.68	−0.209	0.9997
19173_01	160	loss	4	101577666	106585658	DEL	M	5.01	0.68	−0.216	0.9997
19218_01	248	aupd	5	170750020	180837061	UPiD	M	10.09	0.58	0.040	0.9994
20127_01	357	aupd	11	63941311	134433812	UPiD	F	70.49	0.55	0.004	1.0000
20183_01	82	aupd	13	23271930	114108295	UPiD	F	90.84	0.56	0.022	0.9999
21089_01	269	aupd	20	29945359	62207762	UPiD	M	11.09	0.58	−0.019	0.9932
22144_01	75	loss	9	89688030	89910321	NA	F(C)	0.22	0.54	−0.028	1.0000
23020_01	207	loss	18	60033312	75686888	DEL	F	15.65	0.89	−0.614	0.9965

*aupd* acquired UPD; Concordance: the proportion of base pairs in agreement between the regions reported by triPOD and those reported by Laurie *et al*. Additional abbreviations as described in Table 8. Columns 4-11 correspond to the triPOD analysis.

## Discussion

The ability to accurately detect all types of chromosomal abnormalities is vital to advance the understanding of normal and disease processes. Sensitive and specific detection is the first step in uncovering the effects of low-level mosaic alterations on human health. In addition to the role of mosaicism in disease, several groups have hypothesized that the prevalence of large mosaic abnormalities in the brain and liver suggests a role in normal physiological function, possibly associated with a favorable increase in genetic diversity and unique neuronal signaling processes [29-32]. Recent reports have also highlighted a strong correlation between clonal mosaicism and aging, with interesting similarities to cancer [28,33]. We hope that increasingly sensitive techniques for the detection of low-level mosaicism will fuel a surge in mosaicism research. It is also important, for many types of disease research, to be able to detect the presence of large low-level mosaic cell line artifacts in cultured cells [6].

The implementation of the POD method in triPOD greatly increases the ability to detect mosaic abnormalities in SNP array data. Benchmarking with simulated and real mosaic abnormalities reveals the superior sensitivity of the triPOD software. The analysis of large mosaic abnormalities within the AGRE autism dataset reveals that triPOD allows for identification of previously undetected mosaic abnormalities. Since the AGRE samples are derived from transformed cell lines, it is impossible to distinguish large regions of low-level mosaicism originating in the patient from cell line artifacts. However, the ability to detect such regions is crucial since low-level aneuploidy has been proposed as a genetic risk factor for idiopathic autism, as detected using extensive cytogenetic analyses in cultured peripheral blood cells [3]. Since triPOD demonstrates superior performance on simulated cancer data when compared with algorithms designed for cancer sample analysis, including the paired mode of BAFsegmentation, we suggest that a POD trio-based approach modified for cancer research may provide more sensitive detection than paired tumor/normal approaches.

Differences in abnormality detection are expected across microarray chips due to differences in the number/density of probes, the number of CNV-specific probes, DNA preparation and quality, flux in the levels of mosaicism during expansion or passage of individual samples, and the frequent appearance of new mosaic abnormalities in transformed cell lines [6]. In spite of these underlying confounders, triPOD was shown to be robust when applied to HapMap trios across four different Illumina microarray chips. Detected abnormalities were highly concordant between chips with similar numbers of CNV-specific probes. While small abnormalities were most likely to overlap one other dataset, large informative abnormalities were most likely to be detected across all chip types. triPOD showed good concordance of large abnormalities with 48% matching across all four chips and 78% matching three or more chips, given the confounding effects of mosaic flux and new abnormalities (Figure 8). Although triPOD was developed for microarray data from the Illumina platform, automated adjustments for sample-specific levels of quality and variation allow for application to other platforms from which SNP-specific genotypes, allelic ratios, and copy number data can be derived. We also anticipate an adaptation of the POD method for analysis of mosaicism in next-generation sequence data.

A comparison of two large datasets reveals that the presence of CNV-specific probes results in a great increase in detected abnormalities. It was discovered that the AGRE dataset harbored significantly more large informative abnormalities than the cleft lip/palate dataset. While there may be multiple reasons for this difference, the source of DNA offers a likely explanation. Large abnormalities may occur with increased frequency or with neutral or advantageous results in cell culture, resulting in frequent mosaic events due to the process of clonal selection. Additional datasets from cultured and *in vivo* sources will be helpful to identify global patterns resulting from the source of DNA and those resulting from disease-associated processes. triPOD’s findings were highly concordant with all of the previously reported [28] large mosaic abnormalities in the cleft lip/palate dataset. Although software was not released to facilitate benchmarking, it is apparent that triPOD has greater sensitivity than the Laurie *et al*. approach.

Although validation of the newly discovered very low-level mosaic abnormalities by additional experimental approaches would be ideal, the correlation between alterations of the heterozygous BAF band and underlying mosaic abnormalities has been rigorously validated by multiple groups [9,18,34], as noted by Laurie *et al*. [28]. The current validation approaches are largely infeasible for proving false positives for regions < 5% mosaic. We hope that increasingly sensitive detection algorithms will spur the development of new sequence-based validation techniques.

We have several recommendations for triPOD usage. triPOD may be used concurrently with a CNV-specific algorithm, in order to benefit from both types of specialized detection capabilities. Care should be taken when interpreting large abnormalities in commonly variable regions due to a tendency to combine small adjacent abnormalities of the same type. Interesting findings should be graphically investigated until the user has gained expertise with the strengths and weaknesses of the various detection and annotation methods employed by triPOD. Only in very rare instances of consanguineous relationships (offspring from siblings or bilineal relationships) in which a significant portion of the parental genomes are identical, would there be a large reduction in the number of informative SNPs for analysis. To identify such cases we recommend examining the relationship status of trios using a specialized software program such as kcoeff [35].

We believe that many of our algorithmic approaches, including applications of scan statistics, abnormal chromosome detection using k-means clustering and the jump method, CUSUM applied to boundary refinement following region detection, and error rate estimation, may be novel applications for abnormality detection in SNP array data. We expect that such methods can be generalized and of benefit for alternative analyses of genomic data or similar clustering of observed events over time.

## Conclusions

Application of the POD method to trio-based SNP array data provides a highly sensitive and specific means for detecting chromosomal abnormalities, especially low-level mosaicism. Our software implementation, triPOD, outperformed comparable programs when benchmarked with simulated mosaic data. Examples from the AGRE autism dataset in which a progeny chromosome harbored a large low-level mosaic abnormality highlighted the superior performance of triPOD for sensitive detection of mosaic events. triPOD analyses were shown to be robust across multiple types of microarray chips. Significant differences in the abundance of large abnormalities between two large datasets were revealed, likely due to the DNA source. triPOD makes significant advances in reducing the mosaicism detection barrier and increasing the accessibility of mosaicism-directed research.

## Methods

### Datasets

A simulated tumor dilution dataset made available by Staaf *et al*. [8] was adapted and improved for use with triPOD. The dataset is based on Illumina HumanHap550 Genotyping BeadChip data from HapMap sample NA06991, to which 10 simulated abnormalities were added in a series of 21 mosaic states ranging from 0 to 100 percent normal cells in intervals of 5%. For use with triPOD, Illumina HumanHap550-Duov3 data was obtained for the father (NA06993) and the mother (NA06985) of the simulated sample from the NCBI GEO database (accession GSE16912) and converted as described below. Since any non-simulated chromosomal abnormality contains information revealing the parental origin (e.g. an extra copy of the paternal chromosome or loss of the maternal copy), the simulation dataset was improved by an addition of simulated parental origin for each aberrant region. Parental origin was added by assigning a parent to each of the 10 simulated abnormalities, evaluating the heterozygous SNPs for information content, and reflecting the BAF about the 0.5 axis of any informative SNP which was randomly indicating contribution from the opposite parent. The following abnormalities were assigned paternal origin: hemizygous loss Chr 5q22, single copy gain Chr 8q24, hemizygous loss Chr 9p, hemizygous loss Chr 13q13.1, UPD Chr 17p13.1-p12, and UPD Chr 17q. The following abnormalities were assigned maternal origin: UPD Chr 5p, single copy gain Chr 8p, single copy gain Chr 8q24, hemizygous loss Chr 10q23.1-q23.33, and trisomy Chr 12. One of the remaining limitations in the simulated dataset is that the genotypes are unaltered as the mosaicism changes.

Datasets containing HapMap samples run on Illumina microarray chips were obtained from the NCBI GEO database (see Tables 5 and 6). The datasets were separated into individual samples, annotated, and formatted for analysis by triPOD. The genotyping alleles were converted from HapMap format (ATCG) to Illumina format (AB) by simple replacement (AA = AA, TT; BB = CC, GG; AB = AC, AG, TC, TG; -- = NC). Any markers with alternative genotyping combinations (e.g. CG) were discarded due to the increased complexity of performing the Illumina conversion [36].

The cleft dataset is available in dbGaP (accession phs000094.v1.p1).

### Benchmarking with simulated data

Benchmarking analyses were performed on the autosomes of the simulated dataset using the default parameters with the following programs: PennCNV joint version 2011Jun16, paired sample analysis with BAFsegmentation version 1.2.0, PSCN version 1.0 (*.longlist.update1.txt output), and genoCN version 1.0.0. MAD, as part of R-GADA version 0.9-5, was performed with example parameters from the User’s Guide (aAlpha = 0.8, T = 9, MinSegLen = 75) due to an absence of default parameters. triPOD analyses were performed using only the POD detection algorithm with default parameters. The other optional detection methods were excluded using the following flags: --nohd --nomi1 --nopodcr.

### Performance calculations

Sensitivity and specificity calculations were performed as indicated in Staaf *et al*. [8], with one minor correction. Note that the start position for Aberration 9 was incorrectly reported as 22800000, and is actually 22300000. Briefly, sensitivity was calculated using the ratio of the number of true positive modified heterozygous SNPs in each region/(true positives + false negatives). This calculation was performed for each simulated abnormality with the results of each software program. Overall sensitivity was calculated similarly, using the total modified heterozygous SNPs. Specificity was calculated using the ratio of true negative heterozygous SNPs/(true negatives + false positives). The positive predictive value was calculated using the ratio of true positive heterozygous SNPs/total positives. The results were then plotted (Figures 3 and 4) for comparison with Staaf *et al*. and publications with similar plots [8,9,12,22].

### AGRE boundary detection

Boundary estimation, used for sensitivity calculations, was obtained as local minima or maxima provided by a CUSUM-based approach. CUSUM was applied to a subset of BAF values (≥ the median of the chromosome-specific heterozygous BAF values (0.3 – 0.7) and < 0.7). The *k* parameter was calculated as described above (see Boundary refinement). CUSUM was applied to each of the 12 chromosomes harboring low-level mosaic abnormalities and was able to detect an appropriate change-point for each abnormal region boundary.

### Data quality adjustments

Data quality detection and adjustments have an integral role in maintaining high resolution. The number of undetectable single errors was estimated by mutating all genotype combinations of normal inheritance to all possible single error combinations (*Err*) and expressed as the ratio,

$$\frac{\sum \mathrm{Err}}{\sum \mathrm{MI}1}=\frac{90}{24}=3.75,$$

which relates *Err* to the detectable errors (*MI1*). Assuming that the frequency of occurrence of MI1 combinations is an adequate representation of the error frequency, the overall error rate *e* is estimated as 3.75 times the MI1 rate. This is a conservative rate estimate which does not distinguish between technical and biological causes of MI1 calls in normal regions and includes MI1 inference based on BAF (see Methods: Autosomal rate calculations).

Acceptable levels of quality-related variables were determined based on empirical observation of samples with relationship and DNA quality issues. The analysis of a trio with an estimated MI1 rate ≥ 2% (default) or an autosomal NC rate ≥ 3% (default) for any member is halted, due to a high likelihood of a relationship annotation error or DNA quality issues, respectively, which could invalidate the applicability of the statistical model.

BAF and LRR thresholds are calculated both globally and locally for each sample, adjusting for quality-related variation between samples and chromosomes.

### Detection of normal chromosomes

Normal chromosome arms serve as the basis of several parameter and probability calculations. A two-step process is employed to refine global calculations. The SDs of the autosomal heterozygous BAF values are analyzed as described below to remove outlier chromosome arms before calculating the initial BAF thresholds for informative SNP detection. Informative SNPs are then identified, allowing for the calculation of the *scan statistic* for each arm. The *scan statistic*, *S*_*w*_, is defined as the largest number of events (informative SNPs) in a window of size *w*, given *N* number of points (SNPs) [19]. This novel application of the *scan statistic* provides sensitive identification of arms harboring small abnormalities, which are ignored as the BAF thresholds, autosomal rates, and probability calculations are performed using values from normal arms and imputed values for abnormal ones.

In order to distinguish the normal chromosome arms from those harboring an abnormality, k-means clustering (k = 2) is applied to both the heterozygous BAF SDs and to the *scan statistic* of each arm. Cluster results are then evaluated using the jump method [37]. The jump method is a distribution-independent method for determining the optimal number of clusters in a dataset, including a single cluster. Based on rate distortion theory, the jump method detects a “jump” in the properly transformed distortion curve at the optimal number of clusters. The following equations are employed,

$$K^{*}=\arg\max_{K}\;J_{K},$$

$$J_{K}={\hat{d}}_{K}^{Y}-{\hat{d}}_{K1}^{Y},$$

where K^*^ is the optimal number of clusters, *J*_*K*_ are the jumps in the transformed distortion curve, ${\hat{d}}_{K}$ is the distortion (mean squared error) of k-means clusters and is an estimation of the minimum achievable distortion by fitting *K* centers to a dataset (*d*_*K*_), and *Y* is the transformation factor. The recommended value of *Y* is ≤ *p*/2, where *p* is the dimensionality of the dataset. The *Y* value can be modified to prevent over-clustering. We employ the jump method iteratively to identify a single cluster containing the normal values. Initially, the dataset is clustered (k = 2) and transformed using *Y* = 0.5. If K^*^ = 1, the members of the dataset are considered to be normal. If K^*^ ≠ 1, the cluster with the lowest mean is assumed to contain the normal values and is iteratively reclustered and evaluated with *Y* = 0.475 until the K^*^ = 1. The iterative approach allows for filtering of abnormalities with dissimilar sizes and levels of mosaicism. Lowering *Y* increases the likelihood that members of the normal distribution will remain in a single cluster. Outliers 4SDs from the mean of the normal cluster are then removed.

### Autosomal rate calculations

Several probability estimations are dependent upon autosomal rate calculations. For such calculations, we consider adjacent abnormal SNPs to be more likely of biological origin than error or chance and treat non-adjacent abnormal SNPs identically regardless of biological or technical origin. We assume that the non-adjacent abnormal SNPs in normal regions can provide an adequate estimate of the number of abnormal SNPs expected by chance.

### Parental abnormalities

Since the POD method is modeled upon normal inheritance patterns, somatic abnormalities in parental data, such as cell line artifacts, may be identified as a region of abnormal contribution. Heterozygous MI1 calls (e.g. AA, AA, AB), indicate a novel allele in the child and can be due to a genotyping error, a single nucleotide variant in the child, or a parental somatic alteration. The rate of heterozygous MI1 calls should not be elevated in a detected region unless the detected abnormal inheritance was due to a somatic change in a parent. Once again we can view each polymorphic SNP in the region as a Bernoulli trial where success is defined as a heterozygous MI1 call. We apply a one-tailed binomial test to check for deviations from the expected distribution, where *n* = the number of polymorphic SNPs in the region, *k* = the number of heterozygous MI1calls, and *p* = the autosomal heterozygous MI1 rate. Regions for which the p-value falls below a corrected threshold are indicative of a parental abnormality and are removed from the output. Although the current implementation of triPOD reports only abnormalities in the child, the POD algorithm is adaptable to any member of the trio.

## Availability

triPOD is available for download as a command-line version for use on a Unix-like operating system and as a web application [38]. triPOD is licensed under the terms of the GNU General Public License version 3. See the GNU General Public License for more details. The adapted simulation dataset formatted for use with triPOD is available for download [38]. The current version of triPOD is also provided as Additional file 1.

## Abbreviations

AMP: Amplification; BAF: B allele frequency; CNV: Copy number variation; CUSUM: Cumulative sums; DEL: Deletion; FWER: Familywise error rate; HD: Homozygous deletion; HMM: Hidden Markov model; LRR: Log R Ratio; mBAF: Mirrored B allele frequency; POD: Parent-of-Origin-based Detection; PPV: Positive predictive value; SNP: Single nucleotide polymorphism; SD: Standard deviation; UPD: Uniparental disomy; UPhD: Uniparental heterodisomy; UPiD: Uniparental isodisomy.

## Competing interests

The authors declare that they have no competing interests.

## Authors’ contributions

JDB conceived of the POD method, conceived of and implemented the triPOD software program, performed software testing, benchmarking, and data analysis, and drafted the manuscript. BDB assisted with the strategic design and implementation of triPOD, statistical modeling, and algorithm refinement, and helped to draft the manuscript. MDS participated by facilitating the triPOD web application. JP participated in the acquisition of necessary data and helped to draft the manuscript. All authors read and approved the final manuscript.

## Supplementary Material

Additional file 1 triPOD software. Click here for file
